# Change in Tricuspid Valve Function after Transvenous Lead Extraction, Predisposing Factors and Prognostic Roles

**DOI:** 10.31083/j.rcm2506198

**Published:** 2024-05-30

**Authors:** Wojciech Jacheć, Anna Polewczyk, Dorota Nowosielecka, Andrzej Tomaszewski, Wojciech Brzozowski, Dorota Szczęśniak-Stańczyk, Krzysztof Duda, Agnieszka Nowosielecka, Andrzej Kutarski

**Affiliations:** ^1^2nd Department of Cardiology, Faculty of Medical Sciences in Zabrze, Medical University of Silesia in Katowice, 41-808 Zabrze, Poland; ^2^Department of Medicine and Health Sciences, The Jan Kochanowski University, 25-369 Kielce, Poland; ^3^Department of Cardiac Surgery, The Pope John Paul II Province Hospital, 22-400 Zamość, Poland; ^4^Deptartment of Cardiology, Medical University, 20-059 Lublin, Poland; ^5^Department of Cardiac Surgery Masovian Specialistic Hospital, 26-617 Radom, Poland; ^6^Department of Internal Medicine and Geriatrics, The A.Falkiewicz Specialist Hospital, 52-114 Wrocław, Poland

**Keywords:** transvenous lead extraction, complications, lead-associated tricuspid regurgitation, lead-dependent tricuspid dysfunction

## Abstract

**Background:**

Changes in tricuspid valve (TV) function following transvenous lead extraction (TLE) and their impact on long-term survival have not yet been investigated.

**Methods:**

From 3633 patients undergoing lead extraction between 2006 and 2021, TV function before and after TLE was evaluated in 2693 patients.

**Results:**

After TLE, the TV function remained unchanged in 82.36% of patients, worsened in 9.54%, and improved in 8.10%. Abandoned leads (odds ratio, OR = 1.712; *p = *0.044), fibrotic adhesions between leads and TV apparatus (OR = 3.596; *p* < 0.001), or right ventricular wall (OR = 2.478; *p* < 0.001) were predisposed to TV worsening. Non-infectious indications for TLE (OR = 1.925; *p* < 0.001), the severity of tricuspid valve regurgitation (TVR) before TLE (OR = 3.125; *p* < 0.001), and lead encapsulation (OR = 2.159; *p* < 0.001) were predictors of improvement in TV function. Although either worsening or improving TV function had no impact on long-term survival in all patients, decreased TVR severity in the subgroup of patients with initial regurgitation grades 3–4 was associated with a better prognosis (hazard ratio, HR = 0.622; *p = *0.005).

**Conclusions:**

1. Changes in TV function after TLE were observed in 17.64% of patients. 2. Various factors can predispose to lead-related TV changes, although the common denominator in these events is an extensive buildup of scar tissue. 3. Worsening TV function had no impact on survival after TLE. In patients with severe TV dysfunction, reduction in TVR following TLE was associated with a 40% reduction in mortality during a mean follow-up of 1673 days.

## 1. Introduction

The relationship between permanently implanted ventricular leads and tricuspid valve function is multifaceted. Long-term interactions between endocardial leads and valves can result in loss of leaflet mobility, as described in a series of papers on impaired tricuspid valve (TV) function following right ventricle (RV) lead implantation [1, 2, 3, 4, 5, 6, 7, 8, 9, 10, 11, 12]. Another aspect of lead-valve interaction is accidental damage to the TV during transvenous lead extraction [13, 14, 15, 16, 17, 18, 19, 20, 21, 22]. Scar tissue (ST) surrounding the lead and strong attachments to heart structures predispose to severe TV damage [23]. This issue was not considered in older guidelines [24, 25] but was mentioned in the latest ones [26, 27]. Yet another aspect is lead-dependent TV dysfunction (most frequently regurgitation), a well-known consequence of device placement [28, 29, 30, 31]. Removal of the interfering lead results in a varying degree of reduction in tricuspid regurgitation depending on the duration of pathology and tricuspid annulus diameter [28, 29, 30, 31]. Less is known about usually asymptomatic improvement in TV function after transvenous lead extraction (TLE) in patients with undiagnosed lead-dependent TV dysfunction (LDTVD) [20, 31]. Although generally clinically insignificant, improved TV function appears to result from pre-existing mild lead interference with TV leaflets.

The frequency of occurrence and different circumstances of impairment and improvement in TV function following TLE seem interesting because they represent a pre-existing problem of lead-mediated interference of the tricuspid valve. All previous reports on tricuspid valve changes after TLE were based on relatively smaller groups of patients [1, 2, 3, 4, 5, 6, 7, 8, 9, 10, 11, 12, 14, 15, 16, 17, 18, 19, 20, 21, 22]; therefore, the present study was undertaken to explore this issue in a much larger population.

## 2. Goal of the Study

The objectives of the study were to assess the frequency of occurrence and predisposing factors to changes in tricuspid valve function after transvenous lead extraction and their impact on long-term prognosis after TLE.

Our previous publication, “Tricuspid Valve Damage Related to Transvenous Lead Extraction”, showed that lead implant duration and adhesions between the leads and tricuspid apparatus/right ventricular wall are the main factors responsible for TV damage during TLE. Kaplan–Meier analysis revealed no correlation between TV damage and long-term survival. In the present study, we extended the prognostic factor analysis in which we document the reasons for the lack of impact of TVR progression after TLE on long-term survival, and importantly, we demonstrate the prognostic significance of TVR reduction after TLE in the group with severe TV regurgitation before TLE.

## 3. Methods

### 3.1 Study Population

Data from 2693 TLE procedures performed between 2006 and 2021 at three high-volume centers were performed by the same first operator, recently playing the role of a proctor.

### 3.2 Lead Extraction Procedure 

Indications for TLE procedures were defined according to the latest recommendations on managing lead-related complications (Heart Rhythm Society (HRS) 2017 and European Heart Rhythm Association (EHRA) 2018) [26, 27]. The preferred venous access was the implant vein. In some cases (proximal lead ended inside the cardiovascular system lead broken during the extraction), femoral and jugular access was used as appropriate [32]. The first-line technique for lead extraction was non-powered mechanical systems (Byrd dilatators; Cook®). When polypropylene telescoping sheaths appeared ineffective, powered mechanical sheath systems (Evolution, Cook; TightRail Spectranetics/Phillips) were used. In some cases, during femoral access to the femoral workstation with baskets, the Amplatz GooseNeck® Snare kit (Amplatz, USA), and sometimes Byrd dilators, were used to remove free floating leads from its remnants [32].

### 3.3 Echocardiographic Examinations

**Fig. 1.**
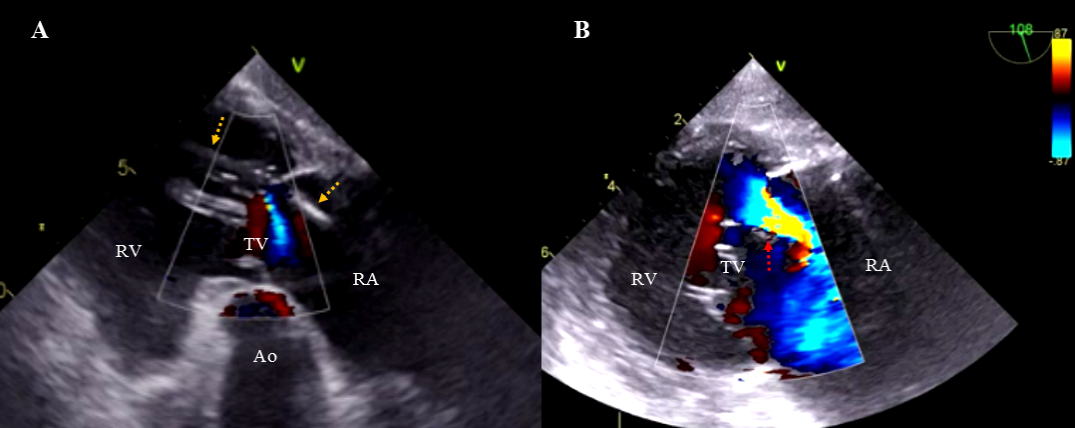
**Tricuspid valve damage—rupture of the chordae tendineae.** (A) Two-dimensional TEE, transgastric projection. Color Doppler. The ventricular lead (yellow arrows) adheres to the posterior leaflet and the sub-valvular apparatus. Mild tricuspid regurgitation, blood leaking backward into the right atrium. (B) Two-dimensional TEE transgastric projection, Color Doppler. Rupture of the chordae tendineae (red arrow) during ventricular lead extraction, worsening regurgitation to a higher grade. RA, right atrium; RV, right ventricle; TV, tricuspid valve; LA, left atrium; Ao, aorta; TEE, transesophageal echocardiography.

**Fig. 2.**
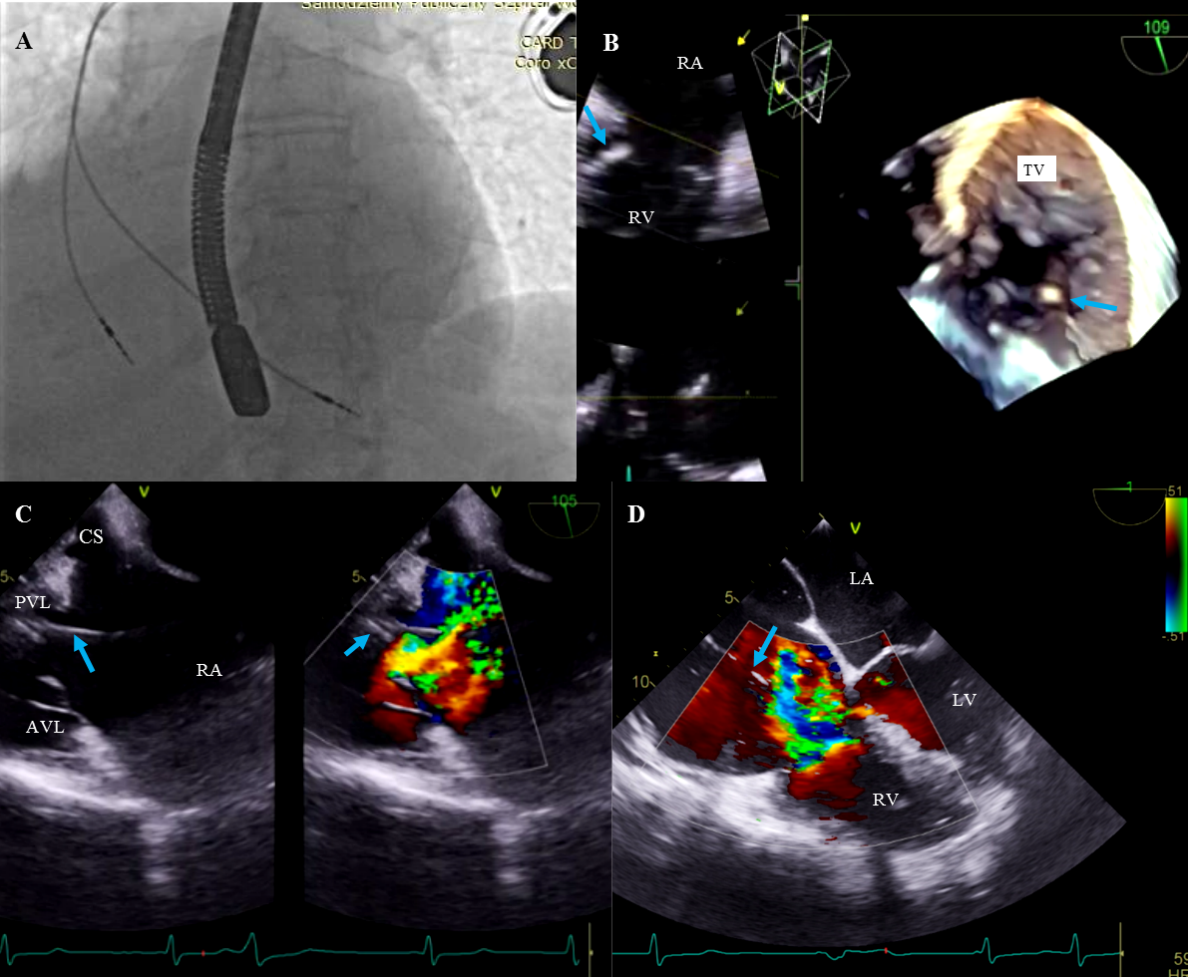
**Lead-related tricuspid valve dysfunction.** (A) Fluoroscopic evaluation of lead positions before TLE procedure. (B) Three-dimensional TEE, mid-esophageal projection. The ventricular lead is impinging on the posterior leaflet (blue arrow). The tricuspid valve is viewed from within the RA during systole. Lack of leaflet coaptation. (C) Two-dimensional TEE, low esophageal projection. Severe tricuspid regurgitation from lead impingement on the posterior leaflet (blue arrow). Dilatation of the RA and TV annulus to 39 mm. (D) Image as in Panel C assessed in the mid-esophageal, four-chamber projection. CS, coronary sinus; PLV, posterior valve leaflet; AVL, anterior valve leaflet; LV, left ventricle; TEE, transesophageal echocardiography; TLE, transvenous lead extraction; RA, right atrium; RV,right ventricle; TV, tricuspid valve; LA, left atrium; Ao, aorta.

**Fig. 3.**
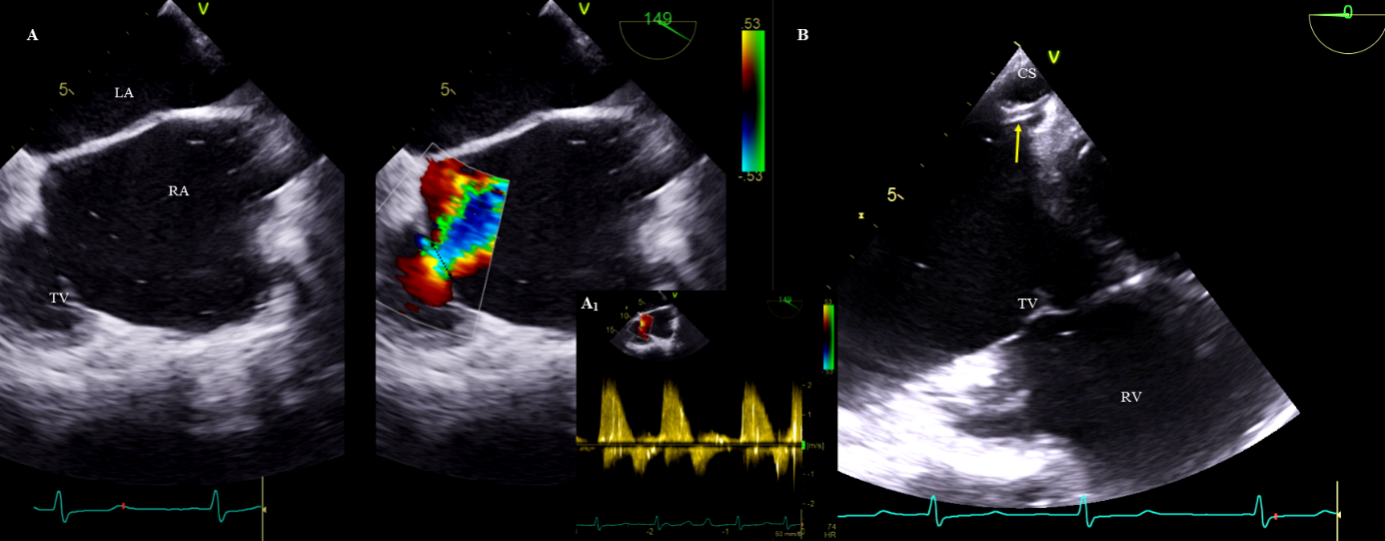
**Evaluation of the tricuspid valve after TLE for massive lead-dependent TV regurgitation.** (A) Two-dimensional TEE mid-esophageal projection, color Doppler. After TLE, the tricuspid valve regurgitation reduction was assessed based on the VC area (compared to Fig. 2—the same patient). (A1) Two-dimensional TEE, CWD. The well-saturated Doppler spectrum of the tricuspid regurgitation with low velocity indicates severe regurgitation of the TV. (B) Implanting a left ventricular lead (yellow arrow) to bypass the tricuspid apparatus. RA, right atrium; RV, right ventricle; CS, coronary sinus; TV, tricuspid valve; LA, left atrium; CWD, continuous wave Doppler; TEE, transesophageal echocardiography; TLE, transvenous lead extraction.

Transthoracic echocardiography (TTE) was mandatory as a pre- and post-procedural examination. Patients with missing echocardiographic examinations were excluded from further analysis. In some patients, continuous transesophageal echocardiography (TEE) was also used to monitor extraction procedures. Mid-esophageal, inferior esophageal, and modified transgastric views were used to visualize the right heart chambers and tricuspid valve. Visualizing multiple anatomical structures and assessing the course of the lead non-standard imaging planes were sometimes required (see Figs. 1,2,3).

All recordings were archived for comparison (pre- and post-operative) of TV and chordae tendineae.

### 3.4 Evaluation of Changes in Tricuspid Valve Function

For the description of changes in TV regurgitation following TLE, standard parameters recommended by the European Association of Echocardiography were used [33]. TR severity was graded using the width of the vena contracta (VC) (semiquantitative parameter) and the color flow area of the regurgitant jet. We used such qualitative parameters as TV morphology, size of the color Doppler jet in relation to right atrium (RA) diameter (grade 1, 2, 3, and 4), and continuous wave (CW) spectral tracing of the regurgitant jet. The additional analysis included all injuries to the sub-valvular apparatus, whereas rupture of the chordae tendineae was regarded as a separate complication.

According to the European Assotiation of Cardiovascular Imaging (EACVI) recommendations, the tricuspid regurgitation severity was graded as mild, moderate, and severe. In mild TR: color flow jet: 1 and 2; CW jet: faint/parabolic; VC ≤3 mm. In moderate TR: color flow jet: 3; CW jet: dense/parabolic; VC >3 and <7 mm. In severe TR: color flow jet: 4; CW jet: dense/triangular with early peaking (peak <2 m/s in massive TR); VC ≥7 mm.

To assess the influence of lead extraction on TV function, an increase in TR by at least one grade was regarded as an impairment, whereas every decrease in TR by at least one grade was considered an improvement in TV function.

### 3.5 Statistical Analysis 

The Shapiro–Wilk test showed that most continuous variables were normally distributed. For uniformity, all continuous variables are presented as the mean ± standard deviation. The categorical variables are presented as numbers and percentages.

Patients were divided into three groups depending on the direction of change in TV function after TLE: group 1: patients without change in TV function; group 2: patients with a reduction in tricuspid regurgitation; group 3: patients with worsening regurgitation.

The significance of the differences between groups was determined using the nonparametric ${\mathrm{Chi}}^{2}$ test with Yates correction or the unpaired Mann–Whitney U test, as appropriate.

For analysis of factors predisposing to impairment or improvement in TV function after TLE uni- and multivariable logistic regression analyses were used. Variables achieving statistical significance (*p* < 0.05) using the Mann–Whitney U test or the ${\mathrm{Chi}}^{2}$ test were included in the univariable model. Any noncorrelated variable with a significant univariable test (*p* < 0.05) was selected for the multivariable analysis.

The proportional Cox regression hazard model was used to determine the impact of change in TVR on survival after TLE. Two models were constructed. In the first one, analysis was performed in all groups of patients. In the second one, patients with grade 3–4 tricuspid regurgitation only were included. All variables (avoiding highly correlated data) having a significant univariable test at *p* < 0.05 were selected for the multivariable regression analysis. To assess the effect of change in TVR on mortality, Kaplan–Meier survival curves were plotted, the course of which was assessed using the log-rank test. A *p*-value less than 0.05 was considered statistically significant. Statistical analysis was performed using Statistica 13.3 (TIBCO Software Inc. Tulsa, OK, USA).

## 4. Results

The study population consisted of 2693 patients, average 66.82 years, 39.44% females, an average left ventricular ejection fraction (LVEF) of 49.44%, renal failure (any) in 20.98%, ischemic heart disease in 58.34%, Charlson comorbidity index of 4.83, systemic infection (with pocket infection or not) in 23.25%, local (pocket) infection in 8.17%, lead failure (replacement) in 50.24%, change of pacing mode/upgrading, downgrading and other in 18.27%, pacemaker (any) in 70.29%, and implantable cardioverter defibrillator (ICD) (any) or cardiac resynchronisation therapy defibrillator (CRT-D) in 29.71% of patients. The mean dwell time of the oldest lead per patient before TLE was 104.8 months, and the mean cumulative dwell time of the leads before TLE was 15.84 years.

The changes in tricuspid valve regurgitation following TLE are summarized in **Supplementary Table 1** (**Supplementary File**). Most patients (2218 pts, 82.36%) had no changes in TV function (efficacy) after TLE. Some patients (257 pts, 9.54%) experienced an increase in tricuspid regurgitation (TR), although significant worsening was relatively rare (11 pts, 0.40%). On the other hand, some patients (218 pts, 8.10%) showed a reduction in TR severity. The improvement was most frequently non-significant (196 pts, 7.28%); only 26 patients (0.97%) had a significant improvement in TV function. It is noteworthy that TR decrease was a random occurrence since it was not found only in patients with pre-operative lead-dependent TV dysfunction. A small proportion of patients (21 pts, 0.78%) with significant procedure-related TV damage met the criteria for surgical repair.

The following tables show the circumstances of non-significant and significant changes in TR after lead extraction.

### 4.1 Potential Patient-Related and Cardiac Implantable Electronic Device (CIED)-Related Predisposing Factors to Changes in TV Function after TLE

The potential patient-related factors predisposing to changes in TV function in subjects with unchanged TR (group 1), patients with reduced TR by 1–3 grades (group 2), and patients with increased TR by 1–3 grades (group 3) after TLE are presented in Table 1.

**Table 1.** **Potential patient-related and CIED-related predisposing factors to changes in TV function after TLE**.

	TVR remained unchanged	TVR decreased by 1–3 grades	TVR increased by 1–3 grades	All patients
	Group 1	Group 2	Group 3	N = 2693
	N = 2218	N = 218	N = 257	
	N (%)	N (%)	N (%)	N (%)
	mean ± SD	mean ± SD	mean ± SD	mean ± SD
		${\mathrm{Chi}}^{2}$/Mann–Whitney U test	${\mathrm{Chi}}^{2}$/Mann–Whitney U test	
		*p*: 2 *vs* 1	*p*: 3 *vs* 1	
			*p*: 3 *vs* 2	
Patient age during TLE (years)	66.74 ± 14.36	68.94 ± 14.51	65.67 ± 15.84	66.82 ± 14.53
		*p* = 0.006	*p* = 0.855	
			*p* = 0.030	
Sex (% of female patients)	852 (38.41)	99 (45.41)	111 (43.19)	1062 (39.44)
		*p* = 0.051	*p* = 0.156	
			*p* = 0.694	
IHD as baseline heart disease	1293 (58.30)	134 (61.47)	144 (56.03)	1571 (58.34)
		*p* = 0.404	*p* = 0.529	
			*p* = 0.042	
NYHA class (I–IV)	1.84 ± 0.68	2.01 ± 0.66	1.59 ± 0.61	1.846 ± 0.675
		*p* = 0.001	*p* = 0.015	
			*p* < 0.001	
LVEF (%)	49.24 ± 15.62	46.97 ± 24.85	53.23 ± 13.03	49.44 ± 15.39
		*p* = 0.024	*p* = 0.010	
			*p* < 0.001	
PASP (mm Hg)	30.47 ± 13.24	39.40 ± 11.85	28.58 ± 12.33	31.00 ± 13.33
		*p* < 0.001	*p* = 0.049	
			*p* < 0.001	
Creatinine (mg/dL)	1.193 ± 0.731	1.204 ± 0,775	1.18 ± 0.786	1.193 ± 0.732
		*p* = 0.833	*p* = 0.788	
			*p* = 0.738	
Long-term anticoagulation	865 (39.00)	121 (55.50)	93 (36.19)	1079 (40.07)
		*p* < 0.001	*p* = 0.419	
			*p* < 0.001	
Charlson comorbidity index (points)	4.83 ± 3.67	5.37 ± 3.86	4.33 ± 3.58	4.83 ± 3.69
		*p* = 0.045	*p* = 0.062	
			*p* = 0.005	
TLE indications: LRIE with or without pocket infection	528 (23.81)	35 (16.06)	63 (24.52)	626 (23.25)
		*p* = 0.012	*p* = 0.861	
			*p* = 0.031	
All non-infectious indications	1502 (67.72)	168 (77.07)	175 (68.63)	1845 (68.51)
		*p* = 0.006	*p* = 0.959	
			*p* = 0.038	
Device type: PM (AAI, VVI, DDD, CRT-P)	1520 (68.53)	156 (71.56)	217 (84.44)	1893 (70.29)
		*p* = 0.398	*p* < 0.001	
			*p* = 0.001	
Abandoned leads before TLE	216 (9.74)	23 (10.55)	46 (17.90)	285 (10.58)
		*p* = 0.791	*p* < 0.001	
			*p* = 0.033	
Number of CIED-related procedures before TLE	1.78 ± 1.05	1.67 ± 0.85	2.23 ± 1.26	1.81 ± 1.07
		*p* = 0.779	*p* < 0.001	
			*p* < 0.001	
Fluoroscopic image suggestive of lead interference with TV	102 (4.60)	13 (5.96)	23 (8.95)	138 (5.12)
		*p* = 0.460	*p* = 0.004	
			*p* = 0.240	
Fluoroscopic image suggestive of lead interference with TV (without loop)	24 (1.08)	13 (5.96)	3 (1.17)	40 (1.49)
		*p* < 0.001	*p* = 0.847	
			*p* = 0.009	
Dwell time of the oldest lead per patient before TLE	100.3 ± 75.18	105.6 ± 70.42	142.9 ± 80.65	104.8 ± 76.43
		*p* = 0.075	*p* < 0.001	
			*p* < 0.001	

TLE, transvenous lead extraction; TVR, tricuspid valve regurgitation; IHD, ischemic heart disease; NYHA, New York Heart Association functional classification; LVEF, left ventricular ejection fraction; CIED, cardiac implantable electronic device; LRIE, lead-related infective endocarditis; PM, pacemaker; AAI, single-chamber pacemaker with the tip of the lead in right atrium; VVI, single-chamber pacemaker with the tip of the lead in right ventricle; DDD, dual-chamber pacemaker; CRT-P, cardiac resynchronization therapy pacemaker; TV, tricuspid valve; SD, standard deviation; PASP, pulmonary artery systolic pressure.

It can be seen from the data that patients in group 2 (improvement) were significantly older, had higher NYHA classifications, higher pulmonary artery systolic pressure (PASP), higher Charlson comorbidity index, more often had noninfectious TLE indications, fluoroscopic images suggestive of lead interference with TV (without loop), and slightly longer cumulative dwell times of the leads. In contrast, in group 3 (worsening), the following predisposing factors were more important: Pacemaker device type, abandoned leads, multiple leads before TLE, numerous CIED-related procedures, redundant lead slack (fluoroscopy), fluoroscopic image suggestive of lead interference with TV (without loop), and longer implant duration before TLE. Additionally, the patients in group 3 were statistically younger than those in group 2.

The procedure complexity defined as procedure duration (sheath-to-sheath time), occurrence of technical problems during extraction (any, one technical problem only, two or more technical problems), lead-to-lead adhesion (intraprocedural diagnosis) does not differ between patients with unchanged TR (1) and those with improvement in TV function (2) (see **Supplementary Table 2**—**Supplementary File**). In contrast, all indicators of procedure complexity, such as procedure duration (sheath-to-sheath), average time of single lead extraction, occurrence of technical problems during extraction (any), and lead-to-lead adhesions were much more common in patients with worsening tricuspid regurgitation (3). More extractions of pacemaker leads and fewer extractions of IDC leads were characteristic of patients with impaired TV function after TLE (3). Such factors as the longer dwell time in the oldest extracted lead and the longer cumulative dwell time in the extracted leads were also more common in patients with impaired TV function after TLE (3). Major complications (any) more often occurred in patients with worsening TV function after TLE. Similarly, complete clinical and procedural success rates were lower in this group of patients (3). There were 802 (29.78%) deaths over 1673 ± 1213 (1–5519) days of follow-up. We demonstrated that impaired TV function after lead extraction did not significantly influence long-term survival, probably because impairment affected patients with better general health (see **Supplementary Table 2**—**Supplementary File**).

### 4.2 Echocardiographic Findings in Patients with and without Procedure-Related TV Damage

Table 2 presents an overview of echocardiographic findings in the study patients. Lack of non-significant tricuspid regurgitation before TLE was more frequent in patients with an aggravation of TVR (3) after TLE (totaling 65.76%). However, it was moderate, significant, and severe in patients with a reduction of TVR (2) after TLE (37.62%, 44.95%, and 16.51%, respectively, totaling 99.08%). Various forms of lead-related scar tissue, such as lead thickening, lead adhesion to tricuspid apparatus, RA, or RV walls, were generally more common in patients with worsening TVR after TLE than a large group of patients with unchanged TV function after lead removal. Fibrous tissue build-up in all forms did not differ between patients with reduced TVR after TLE and the controls.

**Table 2.** **Echocardiographic findings in patients with and without procedure-related TV damage**.

Echocardiographic findings	TVR remained unchanged	TVR decreased by 1–3 grades	TVR increased by 1–3 grades	All patients
	Group 1	Group 2	Group 3	N = 2693
	N = 2218	N = 218	N = 257	
	N (%)	N (%)	N (%)	N (%)
	mean ± SD	mean ± SD	mean ± SD	mean ± SD
		${\mathrm{Chi}}^{2}$/Mann–Whitney U test	${\mathrm{Chi}}^{2}$/Mann–Whitney U test	
		*p*: 2 *vs*. 1	*p*: 3 *vs*. 1	
			*p*: 3 *vs*. 2	
Tricuspid valve regurgitation before TLE (-IV)	1.63 ± 0.86	2.73 ± 0.76	1.29 ± 0.76	1.69 ± 0.91
		*p* < 0.001	*p* < 0.001	
			*p* < 0.001	
Tricuspid valve regurgitation after TLE average (-IV)	1.64 ± 0.87	1.61 ± 0.75	2.59 ± 0.87	1.72 ± 0.90
		*p* = 0.796	*p* < 0.001	
			*p* < 0.001	
TVR moderate (II)	617 (27.82)	82 (37.62)	66 (25.68)	765 (28.41)
		*p* = 0.003	*p* = 0.515	
			*p* < 0.001	
TVR significant (III)	294 (13.26)	98 (44.95)	22 (8.56)	414 (15.37)
		*p* < 0.001	*p* = 0.042	
			*p* < 0.001	
TVR severe (IV)	93 (4.19)	36 (16.51)	0 (0.00)	129 (4.79)
		*p* = 0.003	*p* = 0.002	
			*p* < 0.001	
Any shadows on the leads before TLE				
Scar tissue surrounding the lead	212 (9.56)	35 (16.06)	31 (12.06)	278 (10.32)
		*p* = 0.003	*p* = 0.244	
			*p* = 0.263	
Lead thickening	400 (18.03)	43 (19.73)	70 (27.24)	513 (19.05)
		*p* = 0.579	*p* < 0.001	
			*p* = 0.071	
Lead adhesion to heart structures (any)	304 (13.71)	50 (22.94)	88 (34.24)	442 (16.41)
		*p* < 0.001	*p* < 0.001	
			*p* < 0.001	
Lead adhesion to tricuspid apparatus	77 (3.47)	12 (5.50)	54 (21.01)	143 (5.31)
		*p* = 0.181	*p* < 0.001	
			*p* < 0.001	
Lead adhesion to RA wall	80 (3.61)	16 (7.34)	15 (5.84)	111 (4.12)
		*p* = 0.012	*p* = 0.112	
			*p* = 0.635	
Lead adhesion to RV wall	106 (4.78)	17 (7.80)	50 (19.46)	173 (6.42)
		*p* = 0.075	*p* < 0.001	
			*p* < 0.001	
Lead-to-lead adhesion	184 (8.30)	32 (14.68)	39 (15.18)	255 (9.47)
		*p* = 0.002	*p* < 0.001	
			*p* = 0.982	
Abnormal lead loops visible in preoperative TTE/TEE				
Lead loops in the heart (any)/ECHO	372 (17.77)	42 (19.27)	75 (29.30)	489 (18.17)
		*p* = 0.400	*p* < 0.001	
			*p* = 0.017	
*Loop in TV	82 (3.70)	16 (7.737)	22 (8.63)	120 (4.46)
		*p* = 0.015	*p* < 0.001	
			*p* = 0.750	

TLE, transvenous lead extraction; ECHO, echocardiography; TVR, tricuspid valve regurgitation; RA, right atrium; RV, right ventricle; TTE, transthoracic echocardiography; TEE, transesophageal echocardiography; SD, standard deviation; TV, tricuspid valve; *, incomplete data.

### 4.3 Potential Factors for the Improvement in TV Function after TLE

Univariable regression analysis showed that clinical data, echocardiographic parameters (LVEF, PASP, right ventricle diastolic diameter (RVDD), severity of TVR), CIED-, especially lead-related factors, and lead adhesion to RA or RV or another lead were associated with an improvement in TV function after TLE. However, in the multivariable regression analysis, only non-infectious indications for TLE (odds ratio, OR = 1.925; *p* < 0.001), extraction of at least two leads (OR = 1.677; *p* = 0.006), severity of TVR before TLE (OR = 3.125; *p* < 0.001), and scar tissue around the leads (OR = 2.159; *p* = 0.001) were the strongest predictors of an improved TV function after TLE (see Table 3).

**Table 3.** **Factors for improvement in TV function after TLE**.

	Univariable regression			Multivariable regression		
	OR	95% CI	*p*	OR	95% CI	*p*
NYHA functional class (by one)	1.457	1.190–1.783	<0.001	0.804	0.606–1.066	0.130
LVEF (1% *p*)	0.991	0.982–1.000	0.046	0.993	0.981–1.005	0.249
PASP (1 mm Hg)	1.042	1.032–1.052	<0.001	1.007	0.993–1.021	0.310
AF permanent (yes/no)	2.078	1.548–0.790	<0.001	1.194	0.782–1.823	0.410
Long-term anticoagulation (yes/no)	1.767	1.336–2.335	<0.001	0.983	0.660–1.462	0.931
Charlson comorbidity index (one point)	1.039	1.002–1.078	0.037	1.015	0.970–1.061	0.522
LRIE certain with or without pocket infection (yes/no)	0.523	0.335–0.817	0.004			
All non-infectious indications (yes/no)	1.700	1.219–2.371	0.002	1.925	1.312–2.828	<0.001
Fluoroscopic image suggestive of lead interference with TV (yes/no)	1.906	1.194–3.045	0.007	0.730	0.376–1.415	0.350
Extraction of two and more leads (yes/no)	1.423	1.072–1.891	0.015	1.677	1.152–2.440	0.006
Cumulative dwell time of extracted leads (one year)	1.013	1.003–1.023	0.012	0.989	0.975–1.003	0.132
Lead-dependent TV dysfunction (yes/no)	7.600	5.032–11.48	<0.001			
TVR before TLE (one degree)	3.180	2.726–3.708	<0.001	3.125	2.501–3.906	<0.001
Scar tissue surrounding the lead (yes/no)	1.887	1.264–2.816	0.002	2.159	1.351–3.451	<0.001
Lead adhesion to RV wall (yes/no)	1.809	1.058–3.093	0.030	0.825	0.324–2.102	0.686
Lead adhesion to RA wall (yes/no)	2.221	1.269–3.889	0.005	1.442	0.548–3.794	0.458
Lead-to-lead adhesion (yes/no)	1.984	1.313–2.998	<0.001	1.382	0.587–3.256	0.459
Loop in TV (ECHO) (yes/no)	2.024	1.161–3.530	0.013	1.382	0.587–3.230	0.299

TV, tricuspid valve; TLE, transvenous lead extraction; NYHA, New York Heart Association functional classification; PASP, pulmonary artery systolic pressure; RV, right ventricle; AF, atrial fibrillation; LRIE, lead-dependent infective endocarditis; RA, right atrium; ECHO, echocardiography; OR, odds ratio; LVEF, left ventricular ejection fraction; TVR, tricuspid valve regurgitation.

### 4.4 Potential Risk Factors for the Deterioration of TV Function during TLE

Table 4 provides an overview of potential risk factors for the deterioration of TV function during TLE. Multivariable regression analysis showed that the presence of abandoned leads before TLE (OR = 1.712), lead adhesion to tricuspid valve apparatus (OR = 3.596), and right ventricular wall (OR = 2.478) were the strongest predictors of deteriorated TV function after the procedure. The number of previous CIED-related procedures was on the borderline of statistical significance (OR = 1.191; *p* = 0.068). Deterioration of TV function was less likely in patients with initially higher grades of TV regurgitation (OR = 0.581).

**Table 4.** **Risk factors for deterioration of TV function during TLE**.

	Univariable regression			Multivariable regression		
	OR	95% CI	*p*	OR	95% CI	*p*
Patient age at first implantation (one year)	0.985	0.978–0.992	<0.001	1.007	0.994–1.019	0.306
NYHA functional class (by one)	0.734	0.600–0.896	0.002	1.041	0.773–1.401	0.791
Left ventricular ejection fraction (by 1% *p*)	1.017	1.008–1.027	<0.001	0.994	0.981–1.007	0.367
Charlson comorbidity index (one point)	0.963	0.927–0.999	0.047	0.991	0.942–1.042	0.714
Device type—PM (AAI.VVI. DDD.CRT-P) (yes/no)	2.534	1.781–3.607	<0.001	1.849	0.629–5.434	0.264
Abandoned leads before TLE (yes/no)	1.981	1.401–2.801	<0.001	1.712	1.014–2.890	0.044
Number of CIED-related procedures before TLE (by one)	1.339	1.212–1.479	<0.001	1.191	0.987–1.436	0.068
Lead loop crossing TV or in the ventricle (fluoroscopy) (yes/no)	2.120	1.329–3.381	0.002	1.198	0.432–3.321	0.729
Fluoroscopic image suggestive of lead interference with TV (yes/no)	1.853	1.189–2.889	0.006	1.092	0.428–2.786	0.854
Dwell time of the oldest lead (one year)	1.075	1.056–1.094	<0.001	1.012	0.961–1.065	0.657
Technical problem during TLE (any)	2.291	1.729–3.035	<0.001			
Number of major technical problems (by one)	2.072	1.557–2.757	<0.001	1.068	0.710–1.608	0.751
Lead-to-lead adhesion (intraprocedural diagnosis) (yes/no)	2.696	1.831–3.970	<0.001	1.364	0.776–2.395	0.280
Number of extracted leads per patient (by one)	1.307	1.110–1.538	0.001	1.117	0.778–1.603	0.549
Extraction of ICD leads (yes/no)	0.440	0.309–0.627	<0.001	0.873	0.298–2.554	0.804
Extraction of abandoned leads (yes/no)	2.016	1.381–2.942	<0.001			
Dwell time of the oldest extracted lead (one year)	1.077	1.058–1.096	<0.001			
Cumulative dwell time of extracted leads (one year)	1.033	1.025–1.042	<0.001	1.006	0.979–1.033	0.682
TVR before TLE (I–IV) (one grade)	0.612	0.509–0.735	<0.001	0.581	0.481–0.703	<0.001
Lead thickening (yes/no)	1.714	1.261–2.329	<0.001	0.953	0.621–1.462	0.824
Lead adhesion to heart structures (any) (yes/no)	3.854	2.849–5.212	<0.001			
Lead adhesion to tricuspid apparatus (yes/no)	7.493	5.075–11.06	<0.001	3.596	2.150–6.014	<0.001
Lead adhesion to SVC (yes/no)	2.466	1.474–4.124	0.001	1.263	0.624–2.559	0.516
Lead adhesion to RV wall (yes/no)	4.946	3.398–7.199	<0.001	2.478	1.477–4.160	<0.001
Lead-to-lead adhesion (TEE diagnosis) (yes/no)	2.079	1.422–3.040	<0.001	0.841	0.497–1.423	0.518
Lead loops in the heart (any)/ECHO (yes/no)	2.086	1.553–2.803	<0.001	1.475	0.844–2.580	0.173

TV, tricuspid valve; TLE, transvenous lead extraction; NYHA, New York Heart Association functional classification; PM, pacemaker; AAI, single-chamber atrial pacemaker; VVI, single-chamber ventricular pacemaker; DDD, dual-chamber pacemaker; CRT-P, cardiac resynchronization therapy pacemaker; CIED, cardiac implantable electronic device; ICD, implantable cardioverter defibrillator; TVR, tricuspid valve regurgitation; SVC, superior vena cava; RV, right ventricle; ECHO, echocardiography; OR, odds ratio; TEE, transesophageal echocardiography.

In the univariable Cox regression analysis, there was no impact from TLE-derived deterioration of TV function on survival in this long-term follow-up study (see **Supplementary Table 3**—**Supplementary File**).

### 4.5 Prognostic Factors Affecting Survival after TLE in the Entire Group of Patients and in Subgroups with a TVR Grade >2 before TLE

**Fig. 4.**
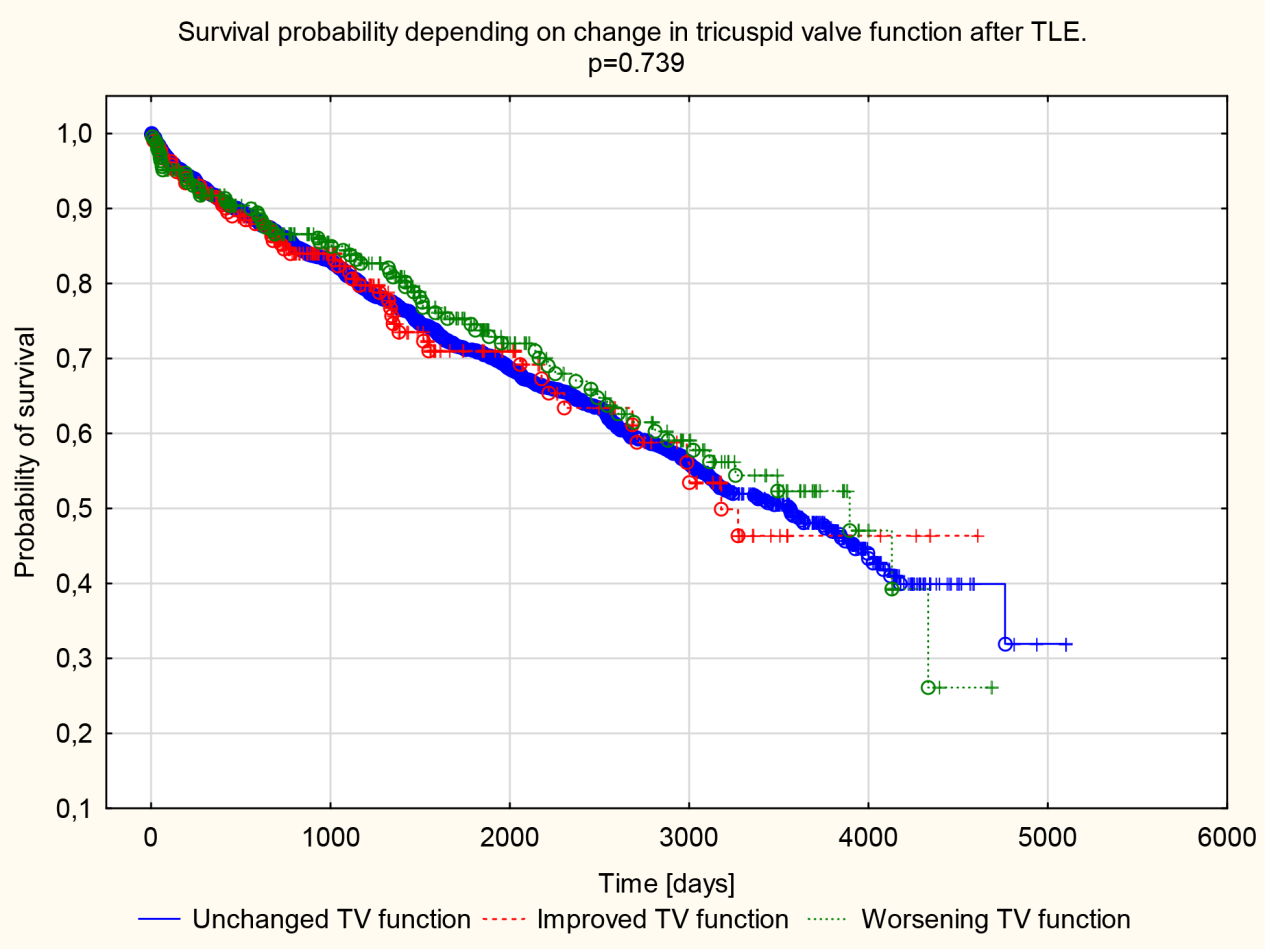
**Kaplan–Meier survival curve.** Survival probability depends on changes in tricuspid valve function after TLE. TLE, transvenous lead extraction; TV, tricuspid valve.

After TLE, 802 (29.78%) patients died during the 1673 ± 1213 (1 – 5100) days of follow-up. Multivariable Cox regression analysis of the entire group of patients confirmed negative effects of the conventional risk factors: Older patient age (hazard ratio, HR = 1.050 per year), higher NYHA class (HR = 1.264 per one), diabetes (HR = 1.317), higher creatinine concentrations (HR = 1.226 per 1 mg/dL), permanent atrial fibrillation (HR = 1.161), higher grade of TV regurgitation (HR = 1.230 per grade), need for implantation of an ICD/CRTD device (HR = 1.314), and infective endocarditis as an indication for TLE (HR = 1.493). Higher LVEF was associated with a better prognosis (HR = 0.976). Neither TV function improvement nor decline had an impact on long-term survival (see Fig. 4). However, multivariable Cox regression analysis of patients with severe TV dysfunction before TLE showed that a decrease in TR severity after TLE by at least one grade was related to a nearly 40% decrease in risk of death in long-term follow-up (HR = 0.622) (see Table 5).

**Table 5.** **Prognostic factors affecting survival after TLE in the entire group of patients and subgroups with a TVR grade >2 before TLE**.

	Univariable Cox regression			Multivariable Cox regression		
	OR	95% CI	*p*	OR	95% CI	*p*
Patient age at first implantation (one year)	1.040	1.034–1.046	<0.001			
Patient age during TLE (one year)	1.045	1.038–1.052	<0.001	1.050	1.043–1.057	<0.001
Female gender (yes/no)	0.667	0.574–0.776	<0.001	0.863	0.749–0.995	0.042
NYHA class (one class)	2.381	2.156–2.630	<0.001	1.264	1.125–1.421	<0.001
Diabetes (yes/no)	1.806	1.541–2.115	<0.001	1.317	1.142–1.519	<0.001
Creatinine (1 mg/dL)	1.420	1.361–1.482	<0.001	1.226	1.165–1.290	<0.001
Ischemic heart disease (yes/no)	1.644	1.420–1.905	<0.001	0.991	0.829–1.184	0.917
Cardiomyopathy (yes/no)	1.556	1.288–1.880	<0.001	1.072	0.843–1.362	0.571
Charlson comorbidity index (by one)	1.139	1.119–1.159	<0.001			
LVEF (1% *p*)	0.963	0.959–0.968	<0.001	0.976	0.970–0.981	<0.001
AF permanent (yes/no)	1.890	1.624–2.199	<0.001	1.161	1.007–1.338	0.039
Tricuspid valve regurgitation after TLE (one grade)	1.504	1.395–1.621	<0.001	1.230	1.144–1.324	<0.001
Tricuspid valve regurgitation before TLE (one grade)	1.556	1.445–1.676	<0.001			
Deterioration of TV function after TLE (yes/no)	0.912	0.716–1.162	0.912			
Improvement of TV function after TLE (yes/no)	1.022	0.777–1.345	0.872			
Infective endocarditis (yes/no)	1.556	1.288–1.880	<0.001	1.493	1.293–1.723	<0.001
Isolated pocket infection (yes/no)	1.218	0.968–1.534	0.093	1.034	0.831–1.238	0.762
Device type before TLE: ICD/CRTD (yes/no)	1.559	1.350–1.802	<0.001	1.314	1.118–1.543	<0.001
Complete procedural success (yes/no)	1.273	0.906–1.787	0.164			
Subgroups of patients with a TVR grade >2 before TLE						
Patient age at first system implantation (year)	1.021	1.011–1.032	<0.001			
Patient age during TLE (year)	1.022	1.010–1.034	<0.001	1.037	1.023–1.051	<0.001
Female gender (yes/no)	0.547	0.415–0.720	<0.001	0.881	0.663–1.170	0.381
NYHA class (one class)	1.818	1.524–2.169	<0.001	1.255	1.024–1.539	0.029
Diabetes (yes/no)	1.899	1.432–2.519	<0.001	1.434	1.090–1.880	0.010
Creatinine (1 mg/dL)	1.225	1.142–1.315	<0.001	1.104	1.017–1.198	0.018
Ischemic heart disease (yes/no)	1.212	0.924–1.591	0.165			
Cardiomyopathy (yes/no)	1.736	1.249–2.412	0.001	0.983	0.695–1.391	0.925
Charlson comorbidity index (by one)	1.119	1.083–1.157	<0.001			
LVEF (1% *p*)	0.965	0.956–0.974	<0.001	0.978	0.967–0.988	<0.001
AF permanent (yes/no)	1.577	1.212–2.053	0.001	1.072	0.831–1.383	0.593
Tricuspid valve regurgitation before TLE (one grade)	1.706	1.253–2.322	<0.001	1.363	1.010–1.839	0.043
Tricuspid valve regurgitation after TLE (one grade)	1.513	1.235–1.853	<0.001			
Deterioration of TV function after TLE (yes/no)	0.738	0.347–1.568	0.429			
Improvement of TV function after TLE (yes/no)	0.573	0.405–0.809	0.002	0.622	0.446–0.870	0.005
Device type before TLE: ICD/CRTD (yes/no)	1.910	1.443–2.528	<0.001	1.503	1.109–2.037	0.009
Infective endocarditis (yes/no)	2.073	1.564–2.747	<0.001	1.538	1.169–2.025	0.002
Isolated pocket infection (yes/no)	1.211	0.772–1.900	0.404			
Complete procedural success (yes/no)	1.297	0.707–2.379	0.401			

TLE, transvenous lead extraction; TVR, tricuspid valve regurgitation; NYHA, New York Heart Association functional classification; LVEF, left ventricular ejection fraction; AF, atrial fibrillation; TV, tricuspid valve; ICD, implantable cardioverter defibrillator; CRTD, cardiac resynchronization therapy defibrillator; OR, odds ratio.

**Fig. 5.**
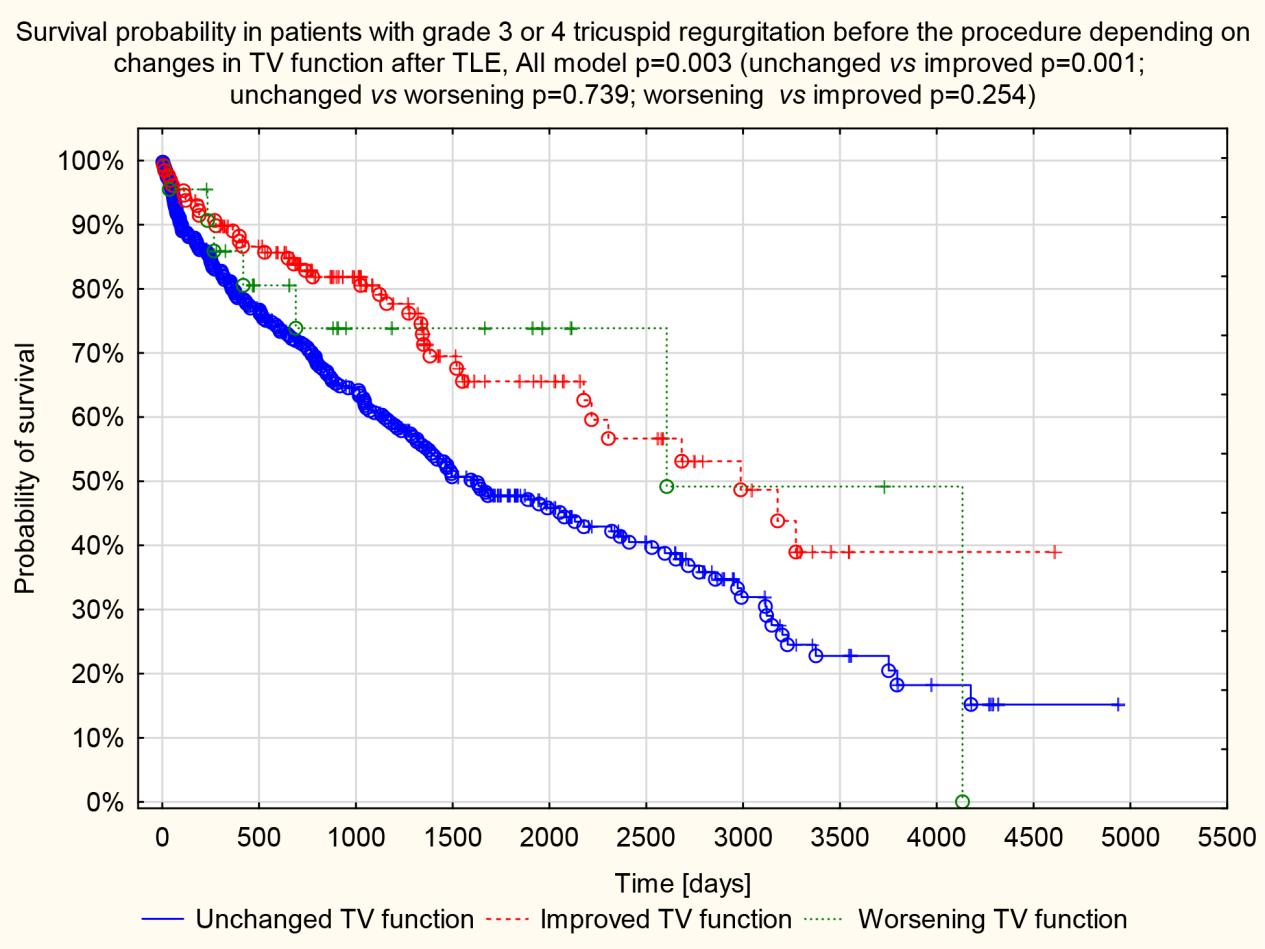
**Kaplan–Meier survival curve.** Survival probability in patients with grade 3 or 4 tricuspid regurgitation before the procedure depends on changes in tricuspid valve function after TLE. TLE, transvenous lead extraction; TV, tricuspid valve.

The log-rank test and the Kaplan–Meyer survival curves confirmed the beneficial effects of improved TV function after TLE in individuals with initially severe TV regurgitation (see Fig. 5).

The statistical significance (all model *p* = 0.003) of the difference in the course of survival curves is determined by the groups: unchanged valve function and with the improvement of valve function (unchanged *vs.* improved *p* = 0.001; unchanged *vs*. worsening *p* = 0.739; worsening *vs*. improved *p* = 0.254).

An increase in the degree of regurgitation from III to IV was observed in 23 people, constituting 4.23% of patients in this group. The worsening of the tricuspid valve regurgitation in the group with an initial TVR >2 degrees (from stage III to IV) did not affect long-term survival, as shown by Cox regression analysis (Table 5, Model 2.); HR = 0.738; 95% CI (0.347–1.568), *p* = 0.429.

## 5. Discussion

The main finding of the study is that TLE unmasks pre-existing lead interference with the tricuspid valve in 17% of patients. Lead removal improves TV function in 8.10% of patients and worsens TR in 9.54%. Interference of RV lead with TV was described in numerous reports in patients after CIED implantation [1, 2, 3, 4, 5, 6, 7, 8, 9, 10, 11, 12, 13, 14, 15, 16, 17, 18, 19, 20, 21, 22]. An extreme type of such interference was referred to as LDTVD [28, 29, 30, 31]. Another consequence of lead interference is scar tissue formation at the lead-leaflet interface. Lead extraction using different tools may cause damage to the leaflets or chordae tendineae [13, 14, 15, 16, 17, 18, 19, 20, 21, 22]. TLE-related TV damage is now considered a major or minor TLE complication, depending on the severity of damage [26, 27]. Only a few reports demonstrate partial improvement in TV function after lead removal in patients with proven LDTVD [4, 20, 31]. The present study, conducted in a large group of patients (2693) before and after TLE, has identified yet another facet of lead-TV interaction—unexpected improvement in TV function. We demonstrated a nonsignificant improvement (by one grade) in TV function in 192 pts (7.13%) and a significant improvement (by 2 or 3 grades) in 26 pts (0.97%). It is clear that an increase in TR severity is a much more important complication: Nonsignificant TVR worsening (by one grade) in 188 pts (6.98%) and significant aggravation of TVR (by 2 or 3 degrees) in 69 pts (2.56%). These results are partly consistent with data obtained in other studies [1, 2, 3, 18, 19, 20, 21, 22], and the differences can be attributed to lead implant duration (age of extracted leads). Reduction in TVR after TLE seems to have important practical consequences and proves that pre-existing undiagnosed lead interference with tricuspid leaflets is more common than expected. In general, deterioration (257 pts, 9.54%) and improvement (218 pts, 8.21%) in TV function following TLE, as a result of previously overlooked lead-valve interactions (lead impingement or adherence to the tricuspid leaflet) appeared to be a relatively frequent phenomenon (475 pts, 17.64%).

As was previously described [34], patients with worsening TV function were more likely to be younger during system implantation, in the lower NYHA classification, without significant or severe TVR, and with a lower Charlson comorbidity index. Multivariable Cox regression showed that worsening TR after TLE was associated with the presence of lead abandonment, degree of TVR before TLE, and lead adhesion to the tricuspid apparatus or right ventricle wall. Deterioration of tricuspid valve function does not affect long-term prognosis after the procedure.

On the other hand, in the group with improving TV function, a multivariable Cox regression analysis did not show that higher NYHA classification, higher PASP, atrial fibrillation presence, need for long-term anticoagulation and higher Charlson comorbidity index were predictors of improvement of TV function after TLE. Among others, the higher degree of tricuspid valve regurgitation before TLE and connective tissue scar surrounding the leads were independent predictors of its improvement. Moreover, a decrease in regurgitation severity after TLE was associated with a better long-term prognosis after lead removal in patients with severe tricuspid regurgitation before TLE.

Moreover, a decrease in regurgitation severity after TLE is associated with a better long-term prognosis after lead removal in patients with severe tricuspid regurgitation before TLE.

It seems that the key role in worsening or improving tricuspid valve function after TLE, apart from the degree of its regurgitation before TLE and system-dependent factors (abandoned leads, number of extracted leads), plays connective tissue structures as scars tissue surrounding the lead and leads adhesions with heart structures. The lead adhesion to the tricuspid apparatus or the right ventricle wall undoubtedly explains the possibility of the TV function deteriorating due to the mechanical impact on the tricuspid valve during TLE. However, the connective tissue scar surrounding the lead may increase its stiffness, resulting in variable support of the tricuspid valve leaflet and regurgitation.

The accumulation of tissue structures on the leads in the form of accretions, scar tissue surrounding the leads, and lead adhesions with other leads or superior vena cava or heart structures depends on the dwell lead time [26]. Older leads are more common in younger patients with a different clinical phenotype than older patients. Younger patients are healthier, while multiple comorbidities (especially kidney dysfunction and diabetes), the younger age of the leads, and the smaller number of abandoned leads, which are more common in the elderly group [35], are factors that may inhibit the body’s connective tissue response to the presence of intracardiac leads [23]. This is reflected in the higher rate of procedural success of TLE in the older patient groups [34]. Moreover, procedure complexity in patients with improved TV function after TLE was similar to that in the control group (patients without changes in TV after TLE). However, patients with impaired TV function after lead removal were likely to have more complex extraction procedures, more risk factors for major complications, actually more major complications, and no clinical and procedural success.

There are a few reports on the improvement in TV function in patients with LDTVD after TLE [20, 31], yet only Rodriguez showed that 30% of patients were found to have TR before TLE that returned to normal valve function during or after the procedure [20]. It should also be emphasized that TV function was not mentioned in the European Lead Extraction ConTRolled (ELECTRa) and the ELECTRa sub-analysis [36, 37, 38].

Another interesting finding in the present study was the confirmation of the involvement of scar tissue (ST) around the lead in the triad: TV-lead–ST. The role of various morphological forms of lead-related fibrosis, such as lead encapsulation, lead thickening, lead adhesion to tricuspid apparatus, RA or RV wall, and other lead, seems unquestionable in partial leaflet release or damage. Other causes of improved TV function after TLE should also be considered, i.e., implantation of a new lead at a different site and routine use of echocardiography to follow the intracardiac route of the new lead.

In the present study, we showed that the degree of tricuspid valve regurgitation before TLE determines the direction of changes in its function after TLE (which is basically obvious: normal function cannot improve more, and severe/massive dysfunction cannot deteriorate more). It is highly significant to document that improving tricuspid valve function (in the group of patients with severe dysfunction before TLE) is an independent factor in improving the long-term prognosis in this group of patients. In turn, the finding of the valve function deteriorating (due to connective tissue adhesion of the leads with heart structures) after TLE does not translate into a long-term prognosis, probably because it occurs in the population of younger, healthier patients (of course, we mean survival in the broadly understood population of CIED patients and not in the general population).

Clinical recommendations resulting from the performed analyses. TLE-related TV damage may be predictable; however, on the other hand, it is difficult to prevent during the lead extraction procedure. Since the experience of the operator and operational team is important, preference for high-volume centers/operators is indicated. TEE monitoring of the TLE procedure may play a key role. Indeed, excessive pooling of the lead was removed, and TEE warned about flap stretching. Unfortunately, the tendinous cord is visible only after it has been broken. Moreover, since the close relation between implant (ventricular lead) duration and risk of TV damage was proven, earlier lead replacement seems to be the optimal solution, especially in young patients. The second risk factor for TLE-related TV damage is the number of extracted leads. Avoidance of superfluous lead abandonment is the second postulate. The third one is the method of lead implantation; leads should be implanted in such a way as to avoid constant dynamic contact of the lead body with the atrium wall and ventricular wall. This means that creating an unnecessary loop of the ventricular lead in the atrium, implanting the lead tip in a septal position, and an apical location should be avoided.

## 6. Conclusions

1. Changes in TV function after TLE were observed in 17.64% of patients.

2. Various factors can predispose to lead-related TV changes, although the common denominator in these events is an extensive buildup of scar tissue.

3. Worsening TV function had no impact on survival after TLE. In patients with severe TV dysfunction, reduction in TVR following TLE was associated with a 40% reduction in mortality during a mean follow-up of 1673 days.

## 7. Study Limitations 

This study has some limitations. A very experienced team performed all procedures, and it may be challenging to reproduce the results in small-volume centers with less experienced operators and teams. All procedures were performed using all types of mechanical sheaths, although not laser-powered sheaths. We examined only the effects of mechanical dilatation and did not know the effects of laser lead extraction on TV damage. It is a retrospective analysis of prospectively collected data between 2006 and 2021. From 2006 to 2014, TTE and TEE were performed before and after TLE, although in 2015–2021, additional TEE monitoring became routine. For technical reasons, effective regurgitant orifice area (EROA) and regurgitant volume (R vol.) were not calculated during TEE.

## Data Availability

The data sets generated and/or analyzed during the current study are available in the repository: http://usuwanieelektrod.pl.

## Supplementary Material

Supplementary material associated with this article can be found, in the online version, at https://doi.org/10.31083/j.rcm2506198.
