# Supplementary material for: Change in Tricuspid Valve Function after Transvenous Lead Extraction, Predisposing Factors and Prognostic Roles
Source: Rev Cardiovasc Med. 2024 May 30;25(6):198. doi: 10.31083/j.rcm2506198 (PMC11270083; doi:10.31083/j.rcm2506198)
Supplement: Supplementary file 1 [file 2153-8174-25-6-198-s1.docx]

Supplementary Table 1. Impact of transvenous lead extraction on tricuspid valve function.

|  | TVR before TLE | TVR after TLE | Number  of patients  N (%) | Change in TVR severity  [0-4] |
| --- | --- | --- | --- | --- |
|  |  |  |  |  |
| No change in TV function; n = 2218 / 2693 (82.36%) | | | | |
|  | 0 | 0 | 33 (1.49) | 0 |
|  | 1 | 1 | 1173 (52.89) | 0 |
|  | 2 | 2 | 624 (28.13) | 0 |
|  | 3 | 3 | 293 (13.21) | 0 |
|  | 4 | 4 | 95 (4.28) | 0 |
| No change in TV function (all) |  |  | 2218 (100.0) |  |
|  |  |  |  |  |
| Improvement in TV function; n = 218 / 2693 (8.10%) | | | | |
|  | 1 | 0 | 7 (3.211) | -1 |
|  | 2 | 1 | 78 (35.78) | -1 |
|  | 3 | 2 | 76 (34.86) | -1 |
|  | 4 | 3 | 31 (14.22) | -1 |
|  | 3 | 1 | 21 (9.63) | -2 |
|  | 4 | 2 | 4 (1.84) | -2 |
|  | 4 | 1 | 1 (0.46) | -3 |
| Improvement in TV function (all) |  |  | 218 (100.0) |  |
|  |  |  |  |  |
| Impairment of TV function; n = 257 / 2693 (9.54%) | | | | |
|  | 0 | 1 | 27 (10.51) | 1 |
|  | 1 | 2 | 87 (33.85) | 1 |
|  | 2 | 3 | 52 (20.23) | 1 |
|  | 3 | 4 | 22 (8.56) | 1 |
|  | 0 | 2 | 3 (1.17) | 2 |
|  | 1 | 3 | 45 (17.51) | 2 |
|  | 2 | 4 | 9 (3.50) | 2 |
|  | 0 | 3 | 1 (0.39) | 3 |
|  | 1 | 4 | 11 (4.28) | 3 |
| Impairment of TV function (all) |  |  | 257 (100.0) |  |

TVR – tricuspid valve regurgitation, TLE – transvenous lead extraction, N – number, TV – tricuspid valve

Supplementary Table 2. Procedure complexity, efficacy, complications, outcomes and long-term mortality after TLE in the study population.

|  | TVR remained unchanged | TVR decreased by 1-3 grades | TVR increased by 1-3 grades | All patients |
| --- | --- | --- | --- | --- |
|  | Group 1  N = 2218 | Group 2  N = 218 | Group 3  N = 257 | N = 2693 |
|  | N (%)  mean ± SD | N (%)  mean ± SD  Chi^2^ / U Mann Whitney test  P: 2 *vs* 1 | N (%)  mean ± SD  Chi^2^ / U Mann Whitney test  P: 3 *vs* 1  P: 3 *vs* 2 | N (%)  mean ± SD |
| Procedure complexity | | | | |
| Technical problems during TLE (any) | 433 (19.52) | 38 (17.43)  *p =* 0.427 | 89 (34.63)  *p <*  0.001  *p <*  0.001 | 560 (20.80) |
| Number of major technical problems | 1.15 ± 0.37 | 1.18 ± 0.39  *p =* 0.485 | 1.30 ± 0.46  *p <*  0.001  *p =* 0.029 | 1.17 ± 0.39 |
| Lead-to-lead adhesion (intraprocedural diagnosis) | 138 (6.22) | 17 (7.80)  *p =* 0.445 | 38 (14.79)  *p <*  0.001  *p =* 0.026 | 193 (7.17) |
| All extracted RV leads (per patient) | 2190  (0.98 ± 0.56) | 234  (1.07 ± 0.54)  *p =* 0.045 | 284  (1.11 ± 0.52)  *p =* 0.010  *p =* 0.771 | 2708  (1.01 ± 0.55) |
| Potential procedure-related risk factors for major complications | | | | |
| Number of extracted leads per patient | 1.65 ± 0.72 | 1.74 ± 0.70  *p =* 0.080 | 1.79 ± 0.83  *p =* 0.002  *p =* 0.469 | 1.67 ± 0.73 |
| Extraction of ICD leads | 654 (29.49) | 63 (28.90)  *p =* 0.918 | 40 (15.56)  *p <*  0.001  *p <*  0.001 | 757 (28.11) |
| Extraction of abandoned leads | 199 (8.97) | 19 (8.72)  *p =* 0.988 | 46 (17.90)  *p <*  0.001  *p =* 0.006 | 264 (9.80) |
| Dwell time of the oldest lead extracted per patient | 98.65 ± 74.09 | 105.5 ± 70.61  *p =* 0.122 | 140.7 ± 80.72  *p <*  0.001  *p <*  0.001 | 103.2 ± 75.42 |
| Cumulative dwell time of extracted leads [years] | 13.38 ± 12.44 | 15.80 ± 13.62  *p =* 0.008 | 20.14 ± 14.66  *p <*  0.001  *p <*  0.001 | 14.22 ± 12.92 |
| Procedure efficacy and complications | | | | |
| Major complications (any) | 22 (0.99) | 3 (1.38)  *p =* 0.853 | 21 (9.73)  *p <*  0.001  *p =* 0.002 | 50 (1.86) |
| Procedure-related death (intra-, post-procedural) | 0 (0.00) | 0 (0.00)  N | 0 (0.00)  N  N | 0 (.00) |
| Complete clinical success | 2186 (98.56) | 216 (99.08)  *p =* 0.743 | 236 (91.93)  *p <*  0.001  *p <*  0.001 | 2638 (97.96) |
| Complete procedural success | 2133 (96.17) | 213 (97.71)  *p =* 0.337 | 225 (87.55)  *p <*  0.001  *p <*  0.001 | 2571 (95.47) |
| Long-term mortality after TLE | | | | |
| Death over 1673 ± 1213  (1 – 5100) days of follow-up | 682 (30.75) | 54 (24.77)  Log rank test  *p =* 0.411 | 66 (25.68)  Log rank test  *p =* 0.965  *p =* 0.451 | 802 (29.78) |

TLE – transvenous lead extraction, TVR – tricuspid valve regurgitation, TV – tricuspid valve, RV – right ventricle

Supplementary Table 3. Effects of the direction of change in tricuspid regurgitation on long-term survival depending on regurgitation grade before TLE; results of univariable Cox regression analysis.

|  |  | Improvement of TV function after TLE | | | Deterioration of TV function after TLE | | |
| --- | --- | --- | --- | --- | --- | --- | --- |
| TV function before TLE | Number  of patients  N (%) | HR | 95%CI | P | HR | 95%CI | P |
| All patients | 2693 (100.0) | 1.022 | 0.777 - 1.344 | 0.878 | 0.912 | 0.716 - 1.162 | 0.458 |
| Lack of TVR | 64 (2.38) |  |  |  | 2.274 | 0.441 - 11.74 | 0.327 |
| TVR ≥ grade 1 | 2629 (97.62) | 1.020 | 0.776 - 1.343 | 0.882 | 0.915 | 0.719 - 1.166 | 0.474 |
| TVR ≥ grade 2 | 1306 (48.50) | 0.736 | 0.555 - 0.975 | 0.033 | 0.917 | 0.637 - 1.319 | 0.639 |
| TVR ≥ grade 3 | 544 (20.20) | 0.573 | 0.405 - 0.809 | 0.002 | 0.738 | 0.347 - 1.568 | 0.429 |
| TVR = grade 4 | 131 (4.86) | 0.502 | 0.264 - 0.953 | 0.035 |  |  |  |

TV – tricuspid valve, TLE – transvenous lead extraction, TVR – tricuspid valve regurgitation
